# The role of surgery in advanced epithelial ovarian cancer

**DOI:** 10.3332/ecancer.2016.666

**Published:** 2016-08-17

**Authors:** María Martín-Cameán, Elsa Delgado-Sánchez, Antonio Piñera, Maria Dolores Diestro, Javier De Santiago, Ignacio Zapardiel

**Affiliations:** Gynaecologic Oncology Unit, La Paz University Hospital, Madrid 28046, Spain

**Keywords:** ovarian cancer, treatment, surgical treatment, cytoreduction, intraperitoneal chemotherapy

## Abstract

Nowadays, the standard management of advanced epithelial ovarian cancer is correct surgical staging and optimal tumour cytoreduction followed by platinum and taxane-based chemotherapy. Standard surgical staging consists of peritoneal washings, total hysterectomy, and bilateral salpingo-oophorectomy, inspection of all abdominal organs and the peritoneal surface, biopsies of suspicious areas or randomised biopsies if they are not present, omentectomy and para-aortic lymphadenectomy. After this complete surgical staging, the International Federation of Gynaecology and Obstetrics (FIGO) staging system for ovarian cancer is applied to determine the management and prognosis of the patient. Complete tumour cytoreduction has shown an improvement in survival. There are some criteria to predict cytoreduction outcomes based on serum biomarkers levels, preoperative imaging techniques, and laparoscopic-based scores. Optimised patient selection for primary cytoreduction would determine patients who could benefit from an optimal cytoreduction and might benefit from interval surgery. The administration of intraperitoneal chemotherapy after debulking surgery has shown an increase in progression-free survival and overall survival, especially in patients with no residual disease after surgery. It is considered that 3–17% of all epithelial ovarian carcinoma (EOC) occur in young women that have not fulfilled their reproductive desires. In these patients, fertility-sparing surgery is a worthy option in early ovarian cancer.

**Core tip:** Complete surgical staging in ovarian cancer is the most important factor to predict longer survival among these patients. Then, with subsequent chemotherapy, the median survival time has doubled up to more than 100 months, making proper surgical training and updating in the newest chemotherapy schemes essential for the survival of women with ovarian cancer. This review will give the reader a comprehensive and updated view of the role of surgery in the management of ovarian cancer.

## Introduction

Ovarian cancer is the seventh most common cancer and the eighth cause of death from cancer among women worldwide (3.6% of cases and 4.6 % of deaths) [1]. Ovarian cancer is the second most common gynaecological malignancy in the USA and the deadliest one with 21,290 estimated new cases and 14,180 deaths in 2015 [2–4]. It is the fourth most common gynaecologic cancer in Europe with an incidence varying from 6 to 14 per 100,000 person-years [5]. This high death rate is due to its indolent course and to the lack of effective screening methods. Up to 60% of epithelial ovarian cancers are diagnosed at an advanced stage (IIB to IV), with a median age at diagnosis of 63 years [4, 6–8]. Advanced-stage ovarian cancer has a 5-year survival rate of 30-55%, while early stages have a 5-year survival of over 80% [4]. It is also known that women with BRCA1 mutation have a 34–46% risk of developing ovarian cancer and those with BRCA2 have a 10–27% of risk. Prophylactic bilateral salpingo-oophorectomy could result in an 80% risk reduction of developing ovarian cancer among these patients [4].

There are several types of ovarian carcinoma, but epithelial ovarian carcinoma (EOC) is the most frequent one, representing 95% of ovarian cancers. The rest of them develop from other ovarian cells (germ cell tumours and sex cord-stromal tumours) [4]. EOC is also the most frequent cause of death from gynaecological neoplasia [5, 8–10]. Its precise causes are not yet well known, but several pathological mechanisms have been suggested, such as differentiation of ovarian surface epithelium or distal fallopian tube cells reaching the ovary during ovulation [9].

It is important to know the spread pattern of ovarian carcinoma. Ovarian cancer arises within the ovarian surface epithelium, grows focally, and ultimately breaches the ovarian capsule and metastasises by exfoliation or throw the abdominal or pelvic lymphatic system. Cancer cells initially implant throughout the pelvis and right paracolic gutter and across the right diaphragm and gastrointestinal organs [10].

Nowadays, the standard management of EOC is the correct surgical staging in early stages and complete tumour cytoreduction followed by platinum and taxane-based chemotherapy in advanced stages [4]. However, if primary cytoreduction seems not possible due to extensive disease or poor patient condition, patients could be treated with neoadjuvant chemotherapy followed by interval debulking surgery and adjuvant chemotherapy.

Standard surgical staging consists of peritoneal washings, total hysterectomy, and bilateral salpingo-oophorectomy, inspection all abdominal organs and peritoneal surfaces, sampling of suspicious areas for biopsy, total omentectomy, and para-aortic lymphadenectomy. After complete surgical staging, the International Federation of Gynaecology and Obstetrics (FIGO) staging system for ovarian cancer is applied to determine the patient management and prognosis. A new ovarian cancer staging system approved since January 2014 is shown in Table 1 [11]. Primary debulking surgery consists on complete macroscopic tumour removal, including splenectomy, diaphragmatic resection, liver resection, intestinal resection or any other abdominal organ affection, if needed to achieve complete cytoreduction [12–13]. The definitions of optimal and suboptimal cytoreduction change in different studies, making comparisons between them difficult. Cytoreduction is divided into three groups related to patient survival: no macroscopic disease, macroscopic disease up to 1 cm and macroscopic disease larger than 1 cm. These three groups could be referred to as complete resection, minimal residual, and gross residual, respectively [14].

There are three types of surgery. Primary cytoreduction, when the higher tumour mass is removed before any other treatment; interval surgery, used in patients who benefit from neoadyuvant chemotherapy before surgical treatment and secondary cytoreduction, the surgery of the cancer recurrence.

Approximately 16–35% of the ovarian cancers, which were presumed to be in the early stages, are upstaged later on [15].

The laparoscopic approach allows surgical staging with less morbidity and mortality than the laparotomy approach. The operative time is longer in the laparoscopy approach than in the laparotomic approach, but patients benefit from the formation of fewer adhesions, shorter in-hospital stay and quicker recovery. These benefits allow the patient to start chemotherapy treatment earlier. The risk of cyst rupture during surgery is no higher with the laparoscopic approach. To avoid tumour dissemination in the port sites, a laparoscopy bag is needed. The laparoscopic approach is more advantageous when fertility preservation is desired because of the lower rate of adhesions [15].

## Primary cytoreduction

Primary debulking surgery followed by platinum and taxane chemotherapy is the standard treatment for advanced EOC with curative intention [16].

Cytoreduction may include a variety of surgical procedures such as bowel resection, especially rectosigmoid (which is necessary in approximately 30–50% of cases of advanced ovarian cancer), diaphragm stripping, peritoneal resection, splenectomy, partial hepatic or pancreatic resection, cholecystectomy, hysterectomy, and salpingo-oophorectomy [4, 12–13, 16–18].

The extension of the procedure performed at the time of primary cytoreductive surgery depends on the location and extent of the disease, surgeon, and medical centre expertise and the patient’s general condition, and comorbidities [2, 16].

Residual tumour after surgery and chemosensibility to platinum therapy are independent survival-related factors [2, 9, 15, 19]. Complete resection at primary debulking surgery is the most important independent prognostic factor in advanced ovarian carcinoma [4, 16, 20–22]. Survival is inversely correlated with residual disease after surgery. Reports show an increase in survival in patients with residual disease under 1 cm [16, 21–24]. Several Gynaecological Oncologic Group (GOG) trials revealed that R0 resection had the longest median overall survival (64 months vs. 29 months in patients with under 1 cm residual disease) [23]. European prospective randomised trials also showed better results in patients with complete cytoreduction (99.1 months in R0 resection vs. 36.2 months for patients with under 1 cm residual disease) [23]. Debulking surgery achieves the removal of poorly vascularised tumour where chemotherapeutic agents have poor access. It also removes chemoresistant clones, which are less susceptible to respond to chemotherapy [2]. The morbidity associated with a debulking surgery does not increase mortality [2]. In fact, as described before, it improves the survival rate.

Patients with residual disease (even less than 1 cm) had worse prognosis than R0 patients, but also patients with a highest preoperative disease burden had shorter progression-free survival and overall survival, compared with those with moderate or low disease burden [6]. This relationship was maintained in the subset of R0 patients. Consistent with other published studies, this analysis showed significant overall survival and progression-free survival benefits for R0 over residual disease < 1 cm. This finding suggests that more aggressive surgery may be warranted if R0 can be achieved. However, even in these cases, the initial disease burden remained a significant prognostic indicator. R0 can be associated in variations in outcome depending on disease burden [6].

Some patients with advanced epithelial ovarian cancer undergo debulking surgery, but complete cytoreduction is not achieved. This leads to an increase in morbidity with no improvement in overall survival. Several meta-analysis in the USA have shown an optimal cytoreduction rate of 42% [23]. Patients with suboptimal resection will not have an improvement in survival but will suffer an increase in mobility [23, 25–26].

There are some criteria to predict cytoreduction outcomes, based on serum biomarkers levels, preoperative imaging techniques, and laparoscopic-based scores [23, 25–26].

The group of Suidan *et al*. [10] identified three clinical and six radiologic criteria associated with suboptimal cytoreduction: age ≥ 60 years (OR 1.32), CA-125 ≥ 500 U/mL (OR 1.47), ASA 3–4 (OR 3.23), retroperitoneal lymph nodes above the renal hilum (including supradiaphragmatic) > 1cm (OR 1.59), diffuse small bowel adhesions/thickening (OR 1.87), periesplenic lesion > 1 cm (OR 2.27), small bowel mesentery lesion > 1 cm (OR 2.28), root of the superior mesenteric artery lesion > 1 cm (OR 2.4), and lesser sac lesion > 1 cm (OR 4.61). Certain factors were more predictive than others. Lesser sac lesions > 1 cm had a predictive value score of 4, significantly higher than other criteria. This suggests that in patients with carcinoma extensive enough to involve the lesser sac, the disease may have already spread to several other anatomic locations as well. Fotopoulou *et al*. [27] identified that patients with primary EOC are more likely to be optimally debulked if the tumour has not expanded in more than four abdominal fields. The fields more related to incomplete debulking surgery are middle abdomen including radix mesenteriand splenic (or left colic) flexure and the upper abdomen in the region of the porta hepatis. Moreover, this study did not show a relation between the CA 125 levels, ascites or the FIGO stage with the resectability of the disease.

There are also CT-based models to predict the likelihood of optimal cytoreduction in patients with advanced ovarian cancer. Nelson *et al* [28] scored CT scans on the basis of cytoreducible or not cytoreducible, on the basis of several criteria: attachment of the omentum to the spleen and disease >2 cm in mesentery, liver surface or parenchyma, diaphragm, gallbladder fossa, suprarenal paraaortic nodes, pericardial nodes, pulmonary, or pleural nodules. This computed tomography (CT) findings predicted surgical outcome with a sensitivity of 92.3% and a specificity of 79.3%. Another CT-based predictive model was proposed by Bristow *et al*. [29]. Thirteen radiographic features were assigned 1 or 2 points: peritoneal thickening, peritoneal implants ≥ 2 cm, small bowel mesentery disease ≥ 2 cm, large bowel mesentery disease ≥ 2 cm, omental disease extension to the stomach, spleen, or lesser sac, extension to pelvic sidewall, parametria, or hydroureter, large-volume ascites (seen on all cuts), suprarenal paraaortic lymph nodes ≥ 1 cm (with 2 points), diaphragm or lung disease ≥2 cm or confluent plaque, inguinal canal disease or lymph nodes ≥ 2 cm, liver lesion ≥ 2 cm on surface or parenchymal lesion of any size, porta hepatic or gallbladder fossa disease ≥ 1 cm, Infrarenal paraaortic lymph nodes ≥ 2 m (with 1 point). A Predictive index score ≥ 4 had the highest overall accuracy, identified patients undergoing suboptimal cytoreduction with a sensitivity of 100%. Dowdy *et al*. [30] found a model based on diffuse peritoneal thickening (defined as peritoneum >4 mm thick in at least two of the following five areas: lateral colic gutters, lateral conalfascia, anterior abdominal wall, diaphragm, pelvic peritoneal reflections) and ascites on at least two-thirds of the CT cuts, with 68% PPV, 52% sensitivity and was associated with a low rate of optimal cytoreduction. Although CT-based predictive models have been demonstrated to be accurate in original cohorts, they have not assessed for external validity. A significant factor affecting prediction is reliance on surgical expertise to achieve R0 resection [31].

A study carried by Hynninen *et al*. [32] showed that compared to CT, PET/CT did not provide additional clinical value to preoperative treatment planning in this prospective study. Neither CT nor PET/CT are sensitive enough for an accurate estimation of disease in the abdominal cavity, particularly in the small bowel mesentery and in the right upper abdomen. PET/CT is more effective for the detection of extra-abdominal metastasis in patients with EOC.

Fagotti *et al*. [33–34] proposed a Predictive Index Value (PIV) based on objective parameters determined during the pre-cytoreduction laparoscopy. With this model, the likelihood that a patient would have a suboptimal surgical result (PPV) is 100% with a PIV≥8. They evaluated several features and gave to them a score: peritoneal carcinomatosis (score 0 for carcinomatosis involving a limited area and surgically removable by peritonectomy; score 2 for unresectable massive peritoneal involvement and with a military pattern of distribution), diaphragmatic disease (score 0 for no infiltrating carcinomatosis and no nodules confluent with the most part of the diaphragmatic surface; score 2 for widespread infiltrating carcinomatosis or nodules confluent with the most part of the diaphragmatic surface), mesenteric disease (score 0 for no large infiltrating nodules and no involvement of the root of the mesentery as would be indicated by limited movement of the various intestinal segments; score 2 for large infiltrating nodules or involvement of the root of the mesentery indicated by limited movement of the various intestinal segments), omental disease (score 0 for no tumour diffusion observed along the omentum up to the large stomach curvature; score 2 for tumour diffusion observed along the omentum up to the large stomach curvature), bowel infiltration (score 0 for no bowel resection was assumed and no miliary carcinomatosis on the ansae observed; score 2 for bowel resection assumed or miliary carcinomatosis on the ansae observed), stomach infiltration (score 0 for no obvious neoplastic involvement of the gastric wall; score 2 for obvious neoplastic involvement of the gastric wall) and liver metastases (score 0 for no surface lesions; score 2 for any surface lesion).

Two studies carried out by the same group [35–36] demonstrated that use of this scoring system is reproducible at other institutions and had no relation to surgeons’ experience.

Recent surgical techniques to approach complete cytoreduction have also been developed. Implants ablation using argon beam coagulator has been reported to be a useful adjunct to traditional surgery. Also, it appears to significantly increase the feasibility of achieving optimal disease status and complete eradication of all visible tumours in patients with macroscopics metastatic ovarian carcinoma [37–39]. The degree of ABC tissue destruction and haemostasis is proportional to the amount of power setting and duration of application. This technique is particularly effective in eradicating all visible tumour located in the gastrocolic ligament, lesser sac, abdominal peritoneum, bowel mesentery, and pelvis [38].

Disease above the diaphragm presents a challenging issue. Diaphragmatic surgery is the main procedure used in upper abdominal cytoreductive surgery. Thoracoscopy-dependent thoracic exploration includes video-assisted thoracoscopy surgery (VATS) and transdiaphragmatic thoracoscopy. Spirtos *et al*. [40] reported data from the Women’s Cancer Centre at the European Society of Gynaecologic Oncology on 57 women undergoing transdiaphragmatic thoracoscopy during surgery for apparent stage IIIC epithelial ovarian cancer with negative chest radiographs or computed tomography scans. Twenty-three (40%) were found to have disease involving either the parietal or visceral pleurae. All with pleural disease had involvement of the diaphragm peritoneum and over 90% of them had positive retroperitoneal lymph nodes. Most of the disease (88%) found above the diaphragm was small (<1 cm) and could be ablated or resected. Terauchi *et al*. [41] performed a prospective study in patients with advanced ovarian cancer with diaphragmatic metastases evaluating transdiaphragmatic thoracoscopic-assisted pleural biopsy and intrathoracic washings. Ten women with stage IIIC ovarian cancer with prominent diaphragmatic lesions were eligible for transdiaphragmatic thoracoscopy, then biopsy of pleural lesions thought to be metastases, and pleural washings and were enrolled. Three of the 10 women (30%) had metastatic lesions and positive cytologyin the thoracic cavity. Two women had positive biopsy results (20%). Two additional women had positive cytology. A total of seven out of the 10 (70%) were up-staged to stage IV.

The group of Yin *et al*. [42] studied the feasibility of transdiaphragmatic thoracic exploration (TDTE) without the use of thoracoscopy. It is indicated in patients with untapped pleural effusion, full-depth diaphracmatic invasion and positive pleural disease on CT.

## Neoadjuvant chemotherapy and interval surgery

Although cytoreduction is the best treatment for the management of advanced ovarian cancer, the are some factors that make it difficult to achieve complete cytoreduction in certain patients. Patients with poor performance status and unresectable disease are candidates for neoadjuvant chemotherapy. However, there are no uniformly validated selection criteria for immediate referral to neoadjuvant chemotherapy [2, 19]

The group of Aletti *et al*. [1], have identified a subgroup of patients in whom the benefits from aggressive debulking do not appear to outweigh the risks. This very high-risk group could be identified by the following three criteria: high tumour dissemination or stage IV, poor performance status (ASA≥3), poor nutritional status (preoperative albumin levels <3.0 g/dL) or age ≥ 75 years.

In this group, the resulting morbidity seems too high to justify aggressive surgical efforts. The median overall survival was only 17 months. In this study, neoadjuvant approach is proposed to be the best option for this small group of patients.

The role of neoadjuvant chemotherapy is to improve the perioperative morbidity and to shrink the tumour to achieve optimal results [43]. In addition, it allows the selection of platinum-resistant patients [19]. A potential problem using chemotherapy before surgery is the formation of fibrosis, which will make the surgical approach more difficult [21].

A meta-analysis including 835 patients showed that neoadjuvant treatment is associated with inferior overall survival compared to initial surgery. Each 10% increase in cytoreduction is associated with an increase in 1.9 months in median survival [19].

Administration of neoadjuvant treatment is not as present in the literature as primary cytoreduction. There are two randomised, controlled, prospective trials conducted by the European Organisation for Research and Treatment of Cancer Research (EORTC) and The Medical Research Council (MRC) Clinical Trials Unit, which show no significant differences in overall survival between the groups with primary cytoreductive surgery and the one with neoadyuvant chemotherapy before surgery. However, patients with complete resection at primary cytoreduction showed an improvement in survival [23]. Vergote *et al*. [28], showed no differences in mortality between the groups that underwent incomplete primary cytoreduction and the one that received neoadjuvant treatment before surgery. The median overall survival (OS) was 29 months and 30 months, respectively. The median progression free survival (PFS) was 12 months for both groups. However, the overall survival was higher in the group who achieved complete primary surgery. The most frequent sites for residual disease after primary or interval surgery are the diaphragm, the abdominal peritoneum and the pelvis (pouch of Douglas, uterus, bladder, rectum, and sigmoid). The decision whether a patient with advanced ovarian carcinoma (stage IIIC or IV) is a better candidate for primary debulking surgery or for neoadjuvant chemotherapy followed by interval surgery, ought to be made according to the patient characteristics, the surgeons experience, complementary tests (CT, serum biomarkers levels) and laparoscopy [23, 25–26], which may provide information about the extension of the disease. Developing and algorithm to predict which patients will achieve a complete cytoreduction will help to improve results and avoid morbidity.

The laparoscopy-based score of Fagotti *et al*. [44] has an important role in the prediction of optimal cytoreduction among women undergoing interval cytoreductive surgery. With a PIV >4, the probability of optimally resecting the disease at laparotomy was equal to 0.

Within the rate of 3-6 cycles, each incremental chemotherapy cycle was associated with a decrease in 40.1 months in median survival, so the surgery ought to be done as early in the treatment programme as possible [19]. Some guides recommend three cycles of chemotherapy. After this, patients undergo surgery, and receive another three cycles after it [16].

One of the major mediators of tumour angiogenesis is VEGF (vascular endotelial growth factor). In fact, the expression of intratumoral VEGF and VEGF receptor (VEGFR) have been associated with a poor prognosis in ovarian cancer. Bevacizumab, a humanised anti-VEGF monoclonal antibody, has shown a significant response in patients with recurrent ovarian cancer with a significant improvement in progression-free survival, especially in patients with poor prognosis [43]. Due to the bleeding risk and impaired wound healing, the manufacturer recommends at least 4 weeks between bevacizumab and any major operative procedures [45].

In conclusion, for patients in which the surgeon could predict that primary cytoreduction will be incomplete, it is better to apply neoadjuvant chemotherapy before surgery because the results are similar to an incomplete primary cytoreduction but morbidity is reduced [21].

## Secondary cytoreduction

Almost 60% of the patients with EOC will have a recurrence. Life expectancy in recurrent ovarian cancer is between 12 and 18 months [44], but it varies depending on the series and the characteristics of the disease [46].

There are four types of patients depending on the time of the recurrence [16]: the ones who progress during the chemotherapy treatment, called platinum-refractory patients; the ones who progress in the first 6 month after the drug treatment, called platinum-resistant patients; women who progress after 12 months of the treatment, the platinum-sensitive patients; and finally women who progress between 6 and 12 months, with and intermedium sensibility to platinum.

The ROVAR score [47] includes four variables and is designed for predicting recurrence after primary treatment with surgical cytoreduction and platinum-based chemotherapy. This four variables are tumour stage at diagnosis, tumour grade at diagnosis, CA 125 serum levels at diagnosis and the presence of residual disease on CT scan after chemotherapy treatment. The ROVAR score has a sensibility and specificity of 94% and 61%, respectively. It is suggested by some researchers as it is not still validated.

The theoretical benefits of surgery are reducing the number of tumour cells so chemotherapy is more likely to be effective, and removal of poor vascularised disease, eliminating pharmacologic sanctuaries [48].

The application of secondary cytoreduction to these patients seems to have a benefit in survival in selected patients. In a retrospective study [49] factors associated with survival after secondary cytoreduction are diagnosis to recurrence interval of 18 months or more [48, 50], complete cytoreduction after secondary surgery and the presence of 1 or 2 recurrence sites or carcinomatosis. Residual disease is also a significant prognostic factor [50]. Age, tumour grade, histology, and tumour size do not have a significant relation with overall survival [48–49, 51]. A median preoperative CA 125 serum level of 56 or more had a borderline significance. The risk of overall death in these patients is two times higher than in patients with serum level under 56. The prognostic importance of the site of the recurrences are not well established [49]. FIGO stage at the time of recurrence is not a prognostic factor [47] except among FIGO stage-I patients [51]. In several studies [48–49], ascites is not a prognostic factor, but Sehouli *et al*. [51] found highly significant differences between patients with no ascites, ascites <500 ml and ascites >500 ml (.8, 21.4, and 3.8 months of survival).

A study by Van de Laar *et al*. [52], in which two predictive models of complete secondary cytoreductive surgery were evaluated, showed that a good performance status and the absence of ascites were two prognostic factors associated with complete secondary surgery. They conclude that more studies are needed before these two predictive models can be applied in daily clinical practice. This study also showed the importance of complete secondary cytoreduction surgery, with a better survival rate in patients with complete resection than in patients who underwent incomplete secondary cytoreductive surgery.

Chi *et al*. [50] give guidelines and selection criteria to select patients for secondary cytoreduction in recurrent, platinum-sensitive EOC. The goal is to achieve less than 0.5 cm residual disease. For operable patients, the selection criteria suggested are as follows: for patients with only one site of recurrence, with a disease-free interval of 6 months, secondary cytoreduction is the best option; for patients with multiple recurrence sites but no carcinomatosis with a disease-free interval of 12 months, secondary cytoreduction must be offered; and, for patients with carcinomatosis who have a disease-free interval of at least 30 months secondary cytoreduction is also beneficial. They do not recommend offering secondary cytoreduction to patients who have a disease-free interval from 6 to 12 months with carcinomatosis. For patients who have multiple sites of recurrence and a disease-free interval from 6 to 12 months or who have carcinomatosis with a disease-free interval of 13 to 30 months, secondary cytoreduction may be considered, and the decision may be individualised based on various factors, such as the exact disease-free interval (closer to 6 or to 30 months), patient age, performance status, overall general medical condition, and the patient’s preferences.

A randomised study [23] with 550 patients with advance ovarian carcinoma, in whom primary cytoreduction was considered to be maximal, did not show an improvement in progression-free-survival or overall survival auditioning secondary cytoreductive surgery to post-operative chemotherapy with paclitaxel plus cisplatin.

Response rate to second line chemotherapy after recurrence for platinum-sensitive patients is 30% or more, while for platinum-resistant patients, the response rate is lower (from 10 to 25 %) [46].

Braicu *et al*. [53] compared primary with secondary cytoreduction. Complete tumour debulking was achieved more often during primary surgery (77% vs. 50%) with equivalent morbidity, but with maximal surgical effort, residual tumour significantly correlates between the two procedures. Residual tumour after primary surgery was related with residual tumour after secondary cytoreduction [51]. Patients with recurrence have significantly higher rates of involvement of the gastric serosa, serosa of small bowel, and mesentery.

As shown, there are heterogeneous opinions and results of different studies. There are two multicentric and international studies, GOG 213 (a phase-III randomised controlled trial of carboplatin and paclitaxel alone or in combination with bevacizumab followed by bevacizumab and secondary cytoreduction surgery in platinum-sensitive, recurrent ovarian, peritoneal primary and fallopian tube cancer) and DESKOP III AGO-OVAR (a randomised trial evaluating cytoreductive surgery in patients with platinum-sensitive recurrent ovarian cancer) that will define the results and indications in this heterogeneous group of patients.

## Conservative treatment and fertility preservation

It is considered that 3–17% of all epithelial ovarian cancers occur in women under the age of 40 [7]. As a result of late parenthood, there are cases of women in reproductive age with ovarian cancer who have not fulfilled their reproductive desires. In order to give a solution to this problem, fertility-sparing treatment has been successfully attempted in selected women with early ovarian cancer. There are no unanimous consensus on which are the criteria for selecting this patients for conservative surgery. According to the ESGO guidelines, patients should fulfil specific characteristics: younger than 40 years old, referred to a tertiary centre, compliant with a close follow-up during and after treatment in order to detect contralateral ovarian recurrence or uterine malignancy, undergo an adequate staging and the anatomical pathology study should be carried out by a designated gynaecologic pathologist. Patients with grade 3 ovarian cancer should not be candidates for conservative surgery.

Fertility-sparing surgery includes unilateral salpingo-oophorectomy on the side of the ovarian tumour and complete staging including peritoneal sampling, pelvic and para-aortic lymph node dissection, and omentectomy [7]. Laparoscopic approach is the most suitable for these patients because it causes fewer adhesions. It is known that the chemotherapy compromises the ovarian function. Carboplatin and paclitaxel are in general less gonadotoxic than other cytostatics [7].

Many studies [7, 15] have shown good obstetrical results after fertility sparing surgery with a conception rate from 60 to 100% and abortion rate under 30%. This fact makes conservative treatment of an early ovarian cancer a worthy option for those women who have reproductive desires.

## Intraperitoneal chemotherapy

Intraperitoneal (IP) chemotherapy is part of the treatment plan in patients with complete cytoreduction of advanced ovarian cancer. The peritoneal cavity is the principal site of disease in ovarian cancer. The reason for intraperitoneal therapy is that in this way, the peritoneum receives sustained exposure to high concentrations of chemoterapics, while normal tissues, such as bone tissue, are relatively spared. IP drug therapy maximise drug delivery to the tumour without increasing the systemic effects of the drug [5, 8].

Armstrong *et al*. compared two groups of patients with stage-III ovarian cancer after optimal debulking cytoreduction. One group received intravenous paclitaxel and cisplatin and the other one received intravenous paclitaxel and intraperitoneal cisplatin and paclitaxel. The median duration of progression-free survival and overall survival was significantly higher in the intraperitoneal therapy group. However, grade 3 and 4 pain, fatigue, and hematologic, gastrointestinal, metabolic, and neurologic toxic effects were also more common in this group. In addition, patients who received intraperitoneal therapy presented worse quality of life before four cycles and three to six weeks after treatment [8]. Tewari *et al*. analysed patients with stage-III EOC or peritoneal carcinoma with no residual disease > 1 cm after surgery. They concluded that the median PFS in patients treated with intravenous carboplatin and paclitaxel followed by IP cisplatin was 25 months compared with 20 months in the group treated with iv paclitaxel and cisplatin (*P* = 0.019). Overall survival was 61.8 months versus 51.4 months, respectively, (*P* = 0.042). Moreover, IP therapy was associated with a 21% decreased risk of progression and a 23% decreased risk of death [54].

Incomplete coverage of the peritoneum through the catheter could cause a suboptimal treatment effect and catheter-related complications. To overcome these disadvantages, intraoperative IP chemotherapy under hyperthermic conditions (HIPEC) is an alternative. It is described that hiperthermia (41°C and higher) has a direct cytotoxic effect mediated by denaturation of proteins and induction of apoptosis. Moreover, it impairs DNA repair and synergises with platinum drugs that induce DNA damage, increases peritoneal tumour drug penetration and inhibit angiogenesis [5].

In a recent randomised study, Spiolitis *et al*. showed that HIPEC significantly prolonged overall survival (26.7 vs. 13.4 months in the group that did not receive HIPEC, *P* = 0.006) [9]. Several studies show that HIPEC is not indicated in all patients with OC but has an acceptable morbidity and provides a survival advantage if appropriate patient selection is performed in a referral centre [9, 5, 54]. Landrum *et al*. showed that the median PFS for patients with stage-III EOC who underwent suboptimal cytoreduction followed by IP paclitaxel/platinum chemotherapy, was 24.9 months (95% CI, 23.0–29.2 months), and median overall survival was 61.8 months (95% CI, 55.5–69.8 months). Patients treated with IV chemotherapy had a PFS of 20.2 months (95% CI, 17.8–23.5 months) with median overall survival of 50.9 months (95% CI, 45.5–58.6 months). The residual disease after the surgery was a predictor for PFS. In patients with residual disease from 0.6 to 1.0 cm, the overall survival and PFS were decreased. In fact, the median PFS of patients treated with IP chemotherapy in no-residual disease was 60.4 months (95% CI 36.9-N/A) and median with overall survival of 127.8 months (95% CI; 84.7-N/A) [55]. Literature shows higher rates of survival in patients treated with IP chemotherapy instead of HIPEC.

## Future challenges

The latest research efforts have focused on studying the molecular biology of ovarian cancer in order to develop therapeutic modalities that may be effective alone or combined with traditional chemotherapy.

There are five main types of ovarian carcinoma based on molecular genetic alterations that account for the 95% of the cases: high-grade serous (70%), endometrioid (10%), clear cell (10%), mucinous (3%), and low-grade serous carcinomas (<5%) [55]. They all have different epidemiology, genetic risk factors, precursor lesions, patterns of spread, molecular events during oncogenesis, response to chemotherapy, and prognosis. The different cellular mechanisms associated with ovarian oncogenesis and progression are the target of this new therapies [55].

As it is described earlier, women with heterozygous BRCA 1 or BRCA 2 mutations have a significantly increased risk of developing ovarian cancer (40% and 18%, respectively). Studies reveal that Poly(ADPribose) polymerase PARP Inhibitors, such as Olaparib, increase chemotherapeutic sensitivity and increase progression median time. PARP inhibitors in combination with standard chemotherapy is a promising therapeutic agent in ovarian cancer in phase-III trial [56]. Other molecular therapy is targeting the PI3K/AKT/mTOR pathway, which upregulation is associated with poor prognosis in ovarian cancer Its inhibition induces apoptosis of tumoural cells. AKT inhibitors, mTOR inhibitors (such us temsirolimus or everolimus) and dual PI3K-mTOR inhibitors are also being studied [57–58].

Both insulin-like growth factor 1 (IGF1) and 2 are growth hormone mediators that have a significant role in cell growth and are overexpressed in ovarian cancer. Anti-IGF1 and anti-IGF2 receptor therapy has demonstrated a decrease of cisplatin and paclitaxel resistance. AMG 479 is a human monoclonal antibody directed against the IGF1 receptor that is being studied in phase-II trials in patients with recurrent platinum-sensitive ovarian cancer [59].

There are other new molecular therapies being developed: antifolate receptor-mediated therapies (ferletuzumab, EC145), death receptor-mediated (conatumumab) and histone deacetylase (HDAC) inhibitors (vorinostat, valproic acid). In addition, antibody-based tumour vaccines and cytokine-based therapies have verified an improvement in host immune activity in order to eradicate cancer cells [60–61].

## Conclusion

In conclusion, the development of modern techniques and the increased number of experienced surgeons will provide more effective therapeutic options to achieve complete cytoreduction in patients with advanced ovarian cancer. Prospective randomized trials are needed in order to obtain uniform validated criteria to select patients for chemotherapy or cytoreduction, and improve the quality of life of these patients.

## Author contributions

Martin-Camean M, Delgado-Sanchez E, Diestro MD, Piñera A, De Santiago J, and Zapardiel I contributed equally to this work. All four designed the topic, performed the search and review of articles, wrote the manuscript, critically reviewed the manuscript and all approved the final version.

## Supportive foundations

None

## Conflict-of-interest statement

The authors declare that there are no potential conflicts of interest.

## Figures and Tables

**Table 1.** FIGO 2014 ovarian cancer staging.

**STAGE I: Tumour confined to ovaries**		
IA	Tumour limited to 1 ovary, capsule intact, no tumour on surface, negative washings	
IB	Tumour involves both ovaries otherwise like IA	
IC: Tumour limited to 1 or both ovaries		
IC1	Surgical spill	
IC2	Capsule rupture before surgery or tumour on ovarian surface	
IC3	Malignant cells in the ascites or peritoneal washings	
**STAGE II: Tumour involves 1 or both ovaries with pelvic extension (below the pelvic brim) or primary peritoneal cancer**		
IIA	Extension and/or implant on uterus and/or fallopian tubes	
IIB	Extension to other pelvic intraperitoneal tissues	
**STAGE III: Tumour involves 1 or both ovaries with cytologically or histologically confirmed spread to the peritoneum outside the pelvis and/or metastasis to the retroperitoneal lymph nodes**		
IIIA: Positive retroperitoneal lymph nodes and/or microscopic metastasis beyond the pelvis)		
IIIA1	Positive retroperitoneal lymph nodes only	
	IIIA1(i)	Metastasis ≤ 10 mm
	IIIA1 (ii)	Metastasis > 10 mm
IIIA2	Microscopic, extrapelvic (above the brim) peritoneal involvement ± positive retroperitoneal lymph nodes	
IIIB	Macroscopic, extrapelvic, peritoneal metastasis ≤ 2 cm ± positive retroperitoneal lymph nodes. Includes extension to capsule of liver/spleen	
IIIC	Macroscopic, extrapelvic, peritoneal metastasis > 2 cm ± positive retroperitoneal lymph nodes. Includes extension to capsule of liver/spleen	
**STAGE IV: Distant metastasis excluding peritoneal metastasis**		
IVA	Pleural effusion with positive cytology	
IVB	Hepatic and/or splenic parenchymal metastasis, metastasis to extraabdominal organs (including inguinal lymph nodes and lymph nodes outside of the abdominal cavity)	
